# Building on safety, feasibility, and acceptability: the impact and cost of community health worker provision of injectable contraception

**DOI:** 10.9745/GHSP-D-13-00025

**Published:** 2013-10-09

**Authors:** Dawn Chin-Quee, John Bratt, Morrisa Malkin, Mavis Mwale Nduna, Conrad Otterness, Lydia Jumbe, Reuben Kamoto Mbewe

**Affiliations:** a FHI 360, Division of Health Services Research, Research Triangle Park, NC, USA; b FHI 360, Division of Research Utilization, Research Triangle Park, NC, USA; c FHI 360, TB CARE I, Lusaka, Zambia; d ChildFund Zambia, Lusaka, Zambia; e Republic of Zambia Ministry of Health, Lusaka, Zambia

## Abstract

**Background:**

A critical shortage of doctors, nurses, and midwives in many sub-Saharan African countries inhibits efforts to expand access to family planning services, especially in rural areas. One way to fill this gap is for community health workers (CHWs) to provide injectable contraceptives, an intervention for which there is growing evidence and international support. In 2009, with approval from the Government of Zambia (GoZ), FHI 360 collaborated with ChildFund Zambia to design and implement such an intervention as part of its existing CHW family planning program.

**Methods:**

The safety of CHW provision of injectable DMPA (depot medroxyprogesterone acetate) was measured by client reports and by a 21-item structured observation checklist. Feasibility and acceptability were measured by interviews with CHWs and a subset of DMPA clients. The impact of adding DMPA to pill and condom provision was assessed by family planning uptake among the clients of trained CHWs from February 2010 to February 2011. Costs were documented using spreadsheets over the period November 2009 to February 2011.

**Results:**

Scores were high on all measures of safety, feasibility, and acceptability. Couple-years of protection (CYP, protection from pregnancy for 1 year) was provided to 51 condom clients, 391 pill clients, and 2,206 DMPA clients. Of the 1,739 clients new to family planning, 85% chose injectable DMPA, while 13% chose pills and 2% chose condoms. Continuation rates were also high, at 63% after 1 year as compared with 47% for pill users. Incremental costs per couple-year were US$21.24 if 50% of users continue with CHW-provided DMPA.

**Conclusion:**

The study affirms that the provision of injectable contraceptives by CHWs is safe, acceptable, and feasible in the Zambian context, with very high rates of uptake in hard-to-reach areas. High continuation rates among clients mean that costs of the intervention can be low when added to an existing community-based distribution program—a finding that is relevant to program replication (now underway in Zambia).

## Abstract (precis)

This project in Zambia contributes to our understanding of the impact of community-based provision of injectables on method choice and uptake and of the costs of adding DMPA to an established community-based family planning program. The project also illustrates the importance of involving stakeholders from the outset, analyzing costs relevant to scale up, and engaging in policy change dialogue not at the end, but rather throughout project implementation.

## Background

Many sub-Saharan African (SSA) countries face critical shortages of doctors, nurses, and midwives.^1^ This deficit inhibits efforts to expand access to family planning services, especially in rural areas, where access to modern contraceptive methods is limited and few trained personnel are available to provide these services.

Task sharing has been employed as a strategy to address this problem by delegating health care tasks that are usually carried out by doctors and nurses to a lower-level provider who is more accessible to the community. For example, the Government of Zimbabwe recently decided to increase access to antiretroviral treatment (ART) by authorizing trained nurses to prescribe drugs and manage patients in care.^2^

Many countries in SSA deploy lower-level cadres in different forms: as government or nongovernment-affiliated, as volunteers or salaried workers, with limited or wide-ranging responsibilities to the communities they serve. Just in the area of family planning, tasks assigned to community health workers (CHWs) can vary. For example, while CHWs in Rwanda are able to resupply clients with both pills and injectables only after a clinical evaluation, in Uganda they are able to initiate and resupply clients with hormonal methods. In Ethiopia, CHWs even insert implants.^3^ Thus, CHWs can play an important role in providing family planning services. Moreover, success in Uganda, Ethiopia, and other SSA countries suggests that the role of CHWs need no longer be limited to distribution of condoms and oral contraceptive pills or referral to higher-level providers.

A growing body of evidence supports the provision of injectable contraceptives by CHWs in hard-to-reach areas.

At a June 2009 technical consultation convened by the World Health Organization (WHO), the U.S. Agency for International Development (USAID), and Family Health International (now FHI 360), 30 technical and program experts from 18 countries reviewed evidence and experiences from programs using CHWs to expand access to injectable contraceptives. These experts concluded, “Given appropriate and competency-based training, CHWs can screen clients effectively, provide DMPA (depot medroxyprogesterone acetate) injections safely and counsel on the side effects appropriately, demonstrating competence equivalent to facility-based providers of progestin-only injectables.”^4^ With the conclusions endorsed by normative bodies such as the International Federation of Gynecology and Obstetrics, the United Nations Population Fund, the International Council of Nurses, and USAID, more countries in SSA initiated pilot studies, began implementing scale-up efforts, engaged in policy change dialogue, or realized policy changes that allow CHWs to provide injectable contraceptives.

More recently, WHO released a set of guidelines that define health worker roles for maternal and newborn health. These guidelines focus on task sharing among various cadres of health care providers to address the critical human resource shortages in many developing countries. Using the latest scientific evidence and the *Grading of Recommendations Assessment, Development and Evaluation* (GRADE) methodology, WHO endorsed the “initiation and maintenance of injectable contraceptives” by lay health workers using a standard syringe, provided that a strong monitoring and evaluation system is in place.^5^

As of August 2012, 13 countries in SSA were undergoing various stages of rolling out CHW provision of injectables.^6^ Different paths were followed, as some governments changed policy first and then conducted pilot studies and scale up, while others began with pilots before considering scale up and policy change.^7^ The impact of CHW-provided injectable contraception has been measured in countries such as Kenya, Madagascar, Malawi, Nigeria, and Uganda, where these programs reported expanded access to family planning services, increased uptake of family planning methods, reduced workload in clinics, and improved method continuation rates among DMPA users.^8^^,^^9^

In Zambia, the process to gain approval for CHW provision of DMPA began with a request from the government to conduct a pilot study in hard-to-reach areas, where staff turnover also presents a significant problem. As in other SSA countries, use of family planning services among rural women in Zambia is relatively low; the contraceptive prevalence rate (CPR) for modern methods is 37%, compared with 48% in urban areas. Similarly, unmet need in rural areas is 28% (19% spacing, 9% limiting) versus 23% (13% spacing, 10% limiting) in urban areas.^10^ In a July 2009 stakeholder meeting to discuss design of the pilot study, government officials and national stakeholders requested measures of program impact to be included, in addition to local confirmation of the safety, feasibility, and acceptability of CHW provision of injectable contraception.

With approval from the Government of Zambia (GoZ), FHI 360 collaborated with ChildFund Zambia, the local affiliate of ChildFund International (formerly Christian Children's Fund) to design and implement an intervention to introduce injectable contraception into ChildFund's existing CHW family planning program. The ChildFund CHW program has been in operation since 1987. In addition to providing family planning services, ChildFund CHWs also deliver health education on personal hygiene and safe motherhood as well as sensitize the community—focusing on men—about family planning. CHWs are both men and women, with varying levels of secondary school education, who have been chosen by community members to provide basic services at the community level. They are volunteers but receive in-kind remuneration in the form of materials and equipment (bicycles, raincoats and boots, t-shirts, bags) and cost-shared animal restocking. Their initial family planning training, conducted over 2 weeks, uses the GoZ curriculum that also includes topics on clients' rights, anatomy and physiology, HIV/AIDS mode of transmission, family life education, male involvement, distribution and storage of commodities, and more. Although they work for ChildFund, these CHWs are affiliated with and supervised by GoZ health center staff (as well as ChildFund staff), from whom they obtain family planning commodities and to whom they submit records for inclusion in the district data management system.

With ChildFund's assistance, we also collected information on additional, or “incremental,” costs of adding injectable contraception to their ongoing CHW provision of condoms and oral contraceptive pills. As programs grapple with limited resources, such information is needed to estimate costs of scaling up and to establish that an intervention provides “value for money.” Thus, this paper not only presents results on the safety, feasibility, and acceptability of CHW provision of DMPA in the Zambian context, but it also focuses on the impact and costs of adding DMPA to an established community-based family planning program.

The study objectives were to:

- Assess CHW ability to provide DMPA injections to clients safely and effectively
- Assess acceptability of, and client and CHW satisfaction with, community-based delivery of DMPA
- Determine the impact of adding DMPA on family planning uptake and the proportion of pill and DMPA users continuing at 6, 9, and 12 months
- Determine incremental cost per couple-year of protection (CYP) of adding injectable contraceptives to the existing CHW program

## METHODS

### Overview

The safety of CHW provision of injectables was measured by DMPA client reports and by a 21-item structured observation checklist (SOC) divided into 2 scales that measured infection prevention (11 items) and injection procedures (10 items). The SOC was used during a clinic-based practicum. Feasibility and acceptability were measured by interviews with CHWs and a subset of their DMPA clients. The impact of adding injectable contraception to pill and condom provision was assessed by family planning uptake among the clients of trained CHWs from February 2010 to February 2011. Costs associated with adding DMPA to CHW-provided family planning services were documented, using spreadsheets, over the period November 2009 to February 2011.

### Training and Data Collection

ChildFund Zambia selected Mumbwa and Luangwa districts—two of ChildFund's hard-to-reach, poor communities with limited access to health care services—to participate in the pilot study. Mumbwa has 34 health centers, 8 of which are in 6 communities served by CHWs involved in the study. Luangwa has 10 health centers and about 26 health units or health posts, 8 of which had CHWs involved in the study.

Preliminary estimates from Zambia's 2010 unpublished census put the population in Luangwa at about 25,000 and the 6-community ChildFund catchment area in Mumbwa at about 50,000 (out of a total district population of 218,328). The 8 health centers in Mumbwa affiliated with ChildFund CHWs are staffed by 17 providers, while the 8 health posts in Luangwa have only 12 staff members, reflecting a severe shortage of health care workers. Also, CHWs outnumbered Ministry of Health (MOH) staff in those catchment areas.

Forty practicing CHWs (20 from each district) affiliated with the 16 health facilities in Mumbwa and Luangwa were trained by master trainers from the MOH to safely provide DMPA injections in addition to the family planning services they already provided. CHWs received 5 days of didactic instruction on determining method eligibility (screening), counseling and informed choice, client referral, and provision of oral contraceptive pills, condoms, and DMPA.* CHWs also completed a 2- to 4-week clinic-based practicum during which 6 or more DMPA injections had to be successfully administered before the CHW was allowed to provide injections unsupervised. We divided the 21-item structured observation checklist used by evaluators into its 2 main components and calculated a total score for safe injections with regard to infection prevention and to injection procedure. Evaluations of the first and last injections given by each CHW constituted the measures of safety.

Study methods included checklists to measure safe provision, interviews with CHWs and clients, and cost analyses.

The training and practicum were conducted on a staggered schedule in Luangwa and Mumbwa between December 2009 and January 2010. By February 2010, all CHWs were certified and their capabilities introduced to their respective communities at an official graduation ceremony involving local leaders and ChildFund representatives. CHWs were then asked to record all provision of condoms, pills, and injectables to their family planning clients for 13 months—distinguishing between new acceptors, switchers, and continuing users of all methods—using ChildFund Zambia's family planning register, modified to include DMPA.

The study team—comprising FHI 360, ChildFund Zambia, and Ministry of Health staff, the Family Planning Technical Working Group (FPTWG),† and other stakeholders—selected the following measures of impact:

1. Family planning method uptake (expressed in couple-years of protection or CYPs‡)
2. Proportion of CHW clients who are new family planning and new DMPA acceptors
3. Indicators of family planning method continuation by CHW pill and DMPA clients
4. Comparisons between CYPs provided by study CHWs and CYPs recorded by District Health Offices (DHOs)

Approximately 9 months into data collection, we interviewed all CHWs and a subset of their DMPA clients (n = 253) who received their first injection from a CHW between February and April 2010. By then, CHWs had performed several months of DMPA administration, and enough time had elapsed for these initial DMPA users to receive up to 3 injections from a CHW.

ChildFund Zambia also selected 6 male employees—3 assigned to each district—to personally retrieve family planning data on a monthly basis from the 40 CHWs. These men (given bicycles for transportation) were engaged specifically to cover the distances to and from CHWs' homes, discuss and verify data with the CHWs, and transfer the required information from the family planning register to the data retrieval form developed for the study. This form collected information on:

- Method received/used on the first visit in study period
- Whether the client was a continuing user or new family planning acceptor§
- Previous method used
- Number of pill cycles or condoms distributed and the scheduled re-injection date for DMPA clients over the course of the 13-month data collection period

The 6 data retrievers received instruction from the Lusaka-based project coordinator on use of the data retrieval form and the family planning client register. Data retrievers also met with the project coordinator monthly for the first half of data collection, then every 2 to 3 months thereafter to verify the accuracy of the information and to submit CHW family planning uptake data from February 2010 to February 2011.

We also obtained family planning statistics for pills, condoms, and DMPA distribution from the District Health Offices in Luangwa and Mumbwa for the same 13-month time period. These statistics reportedly included both health facility and CHW provision of family planning methods. All analyses were performed with SAS 9.2.^11^

### Assessment of Incremental Costs of Adding DMPA to the CHW Program

The approach to costing the addition of a health service intervention to an existing program is to concentrate on additional or “incremental” costs only, since costs of the existing program would have been incurred even without the intervention. Incremental costs of an intervention can be classified according to 3 phases: 1) planning/ designing the intervention; 2) preparing for service delivery; and 3) delivering the new or improved services. Each phase comprises a set of activities, and each activity uses resources, such as time of trainers and providers and medical supplies and equipment. When costs are attached to resources used in the intervention, the total incremental cost of the intervention may be calculated. Stakeholders may be more interested in costs of activities relevant to scaling up, giving less emphasis to costs in the planning and design phase. In our study, these activities included the following:

1. The training of trainers (ToT) workshop that trained 10 trainers, of which 4 were used for the CHW training courses in Luangwa and Mumbwa
2. The CHW training consisting of 2 workshops, each of which trained 20 CHWs
3. The CHW practicum, which included trainee meal allowances and transport refunds for CHW visits to clinic facilities for mentoring and practice in injection technique
4. Supervision of CHWs, which took place in 2 stages: an intensive initial stage where interactions were more frequent and longer in duration and a second phase in which supervisors checked in with CHWs during routine site visits
5. Overall intervention management by ChildFund staff, who oversaw all activities related to the intervention
6. DMPA commodities, including vials, syringes, cotton, soap, and sharps boxes

We used key informant interviews, record reviews, and periodic progress reports to identify all intervention-related activities and resources, and we designed Excel-based spreadsheets to organize information on the costs of these resources. Most costs reflect actual expenditures, except for personnel costs, which were estimated using MOH salary scales for positions considered to be equivalent to those of research and project staff who implemented the intervention.

Incremental cost per CYP of CHW provision of DMPA was calculated by dividing the annualized incremental cost of the intervention (that is, costs adjusted for the period of the study) by the number of CYPs attributable to the CHW intervention. This indicator provides a sense of the value of resources needed to protect 1 couple from pregnancy for a year through DMPA provided by CHWs in ChildFund's program.

FHI 360's Protection of Human Subjects Committee and ERES Converge Ethical Review Board in Zambia reviewed and approved this study.

## RESULTS

### Safety of DMPA Provision by CHWs

On the 11-item scale on infection prevention procedures, CHWs initially carried out about 9 of the items, or 82%, on average. At their final assessment, the average score increased to a perfect 11, or 100%. Thus, there was improvement from the first to the last DMPA injection evaluated during the practicum, but the starting point was high to begin with.

**Figure 1.**
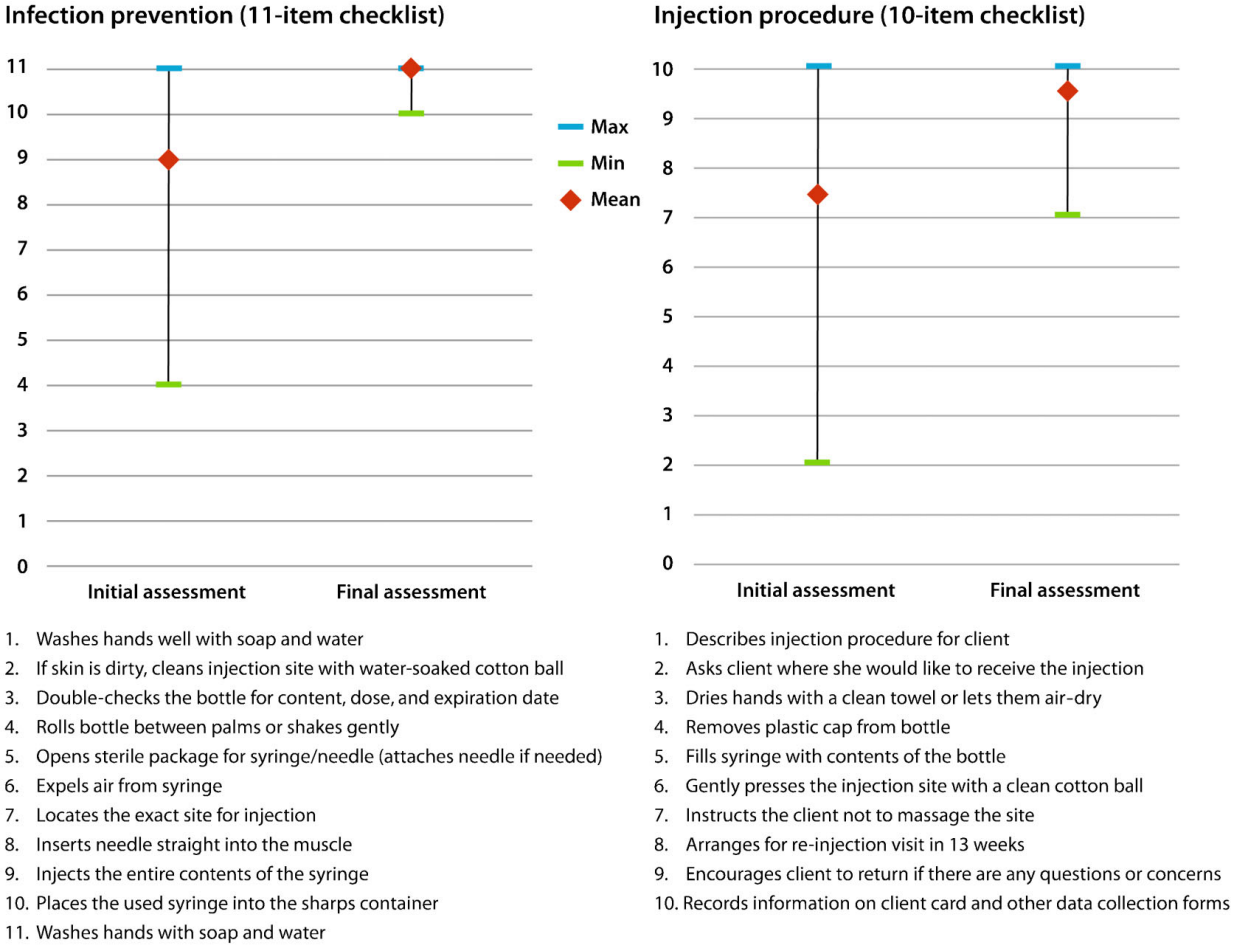
Safety of CHW Provision of Injectables Abbreviations: CHW, community health worker.

For the 10-item injection procedure scale, the initial average score was 7.4, and at final assessment the score was 9.6. Again, there was improvement, but it is important to note that CHWs who scored low in the initial assessment improved markedly by the last assessment. This is captured by the narrowing of the range of scores from the initial to the final assessments for both infection prevention and injection procedure (Figure 1).

Only 6 (2%) of the 253 DMPA clients interviewed after 9 months of data collection reported any problems with CHW-provided injections. Three could not describe the problem, 2 reported pain at the injection site, and 1 client described numbness in the arm. There were no reports from clients of abscesses or infections. During monitoring and supervision activities conducted by ChildFund throughout the study period, CHWs also reported to ChildFund and MOH supervisors that they had not found any abscesses or infections.

### Acceptability of Method to Clients

The acceptability of DMPA and CHW-provision of the method was ascertained by interviews with 253 clients:

- 94% were very much satisfied with DMPA as a family planning method
- 93% wanted to get another DMPA injection
- 94% of CBD clients who accepted DMPA between February and April 2010 received a second DMPA injection
- 99% wanted to receive their next injection from a CHW
- 98% were satisfied with the way the CBD agent gave them their injection
- 98% would recommend receiving a DMPA injection from their CBD agent to a friend

Of the 7% of DMPA users who did not want another injection, the main reasons mentioned were desire to have a child (35%), side effects or problems with DMPA use (35%), and husband's disapproval (18%). For the 4 women (1.6%) who could have but did not receive a second injection, the reasons were dissatisfaction with side effects (n = 2) and husband's disapproval (n = 2). Thus, CHW provision of DMPA and the method itself proved highly acceptable.

In interviews with the 40 CHWs, 98% reported that it was easy to find women who were interested in receiving DMPA, and 80% felt that it was easy to gain the confidence of the community in their ability to provide DMPA. Although 89% said that their workload increased due to the addition of DMPA to their family planning services, they reported that the increase was acceptable and not a burden.

### Characteristics of Acceptors and Family Planning Uptake

Data on family planning method uptake were recorded for a total 4,241 family planning clients in both districts during the 13-month data collection period. The average age of women was 28, with a range of 15 to 53 years. Clients had an average of 3.6 living children, with a range of 0 to 14 children.

Based on provision of methods by ChildFund CHWs from February 2010 to February 2011, 51 condom clients, 391 pill clients, and 2,206 DMPA clients would be protected from pregnancy for 1 year. Uptake of condoms, pills, and DMPA was greater in Mumbwa than Luangwa, as the majority of family planning clients (73%) were from Mumbwa district, the more populous area, with a higher contraceptive prevalence rate (40% vs. 27%).^10^ In both districts, condoms conferred the fewest CYPs, while DMPA conferred the most (Table 1).

Uptake results showed an overwhelming preference for injectable DMPA over pills and condoms.

**Table 1.** Couple-Years of Protection (CYP) by CHW-Provided Methods in Study Districts, February 2010 to February 2011

	**Districts**		
**CYP provided by:**	**Luangwa**	**Mumbwa**	**Total**
Condoms	13	38	**51**
Oral contraceptive pills	96	295	**391**
Injectable DMPA	727	1,479	**2,206**
**Total**	**836**	**1,812**	**2,648**

Source: FHI 360 Data Retriever Registers

Abbreviation: CHW, community health worker; DMPA, depot medroxyprogesterone acetate.

With regard to new acceptors, 41% of CHW clients during the 13-month period were new to family planning. In this group of 1,739 women, 85% chose DMPA, 13% chose oral contraceptive pills, and the remaining 2% chose condoms as their first family planning method. Of the continuing users, 63% reported using pills, 30% DMPA, 6% condoms, and the rest unspecified. Of all the family planning clients in the study, 82%, or 3,479 women, obtained DMPA from a CHW some time between February 2010 and February 2011. About 20% were continuing clients who were formerly clinic clients, and 24% were former pill and condom users who switched to CHW-provided DMPA.

### Continuing Method Use for Pills and DMPA

**Figure 2.**
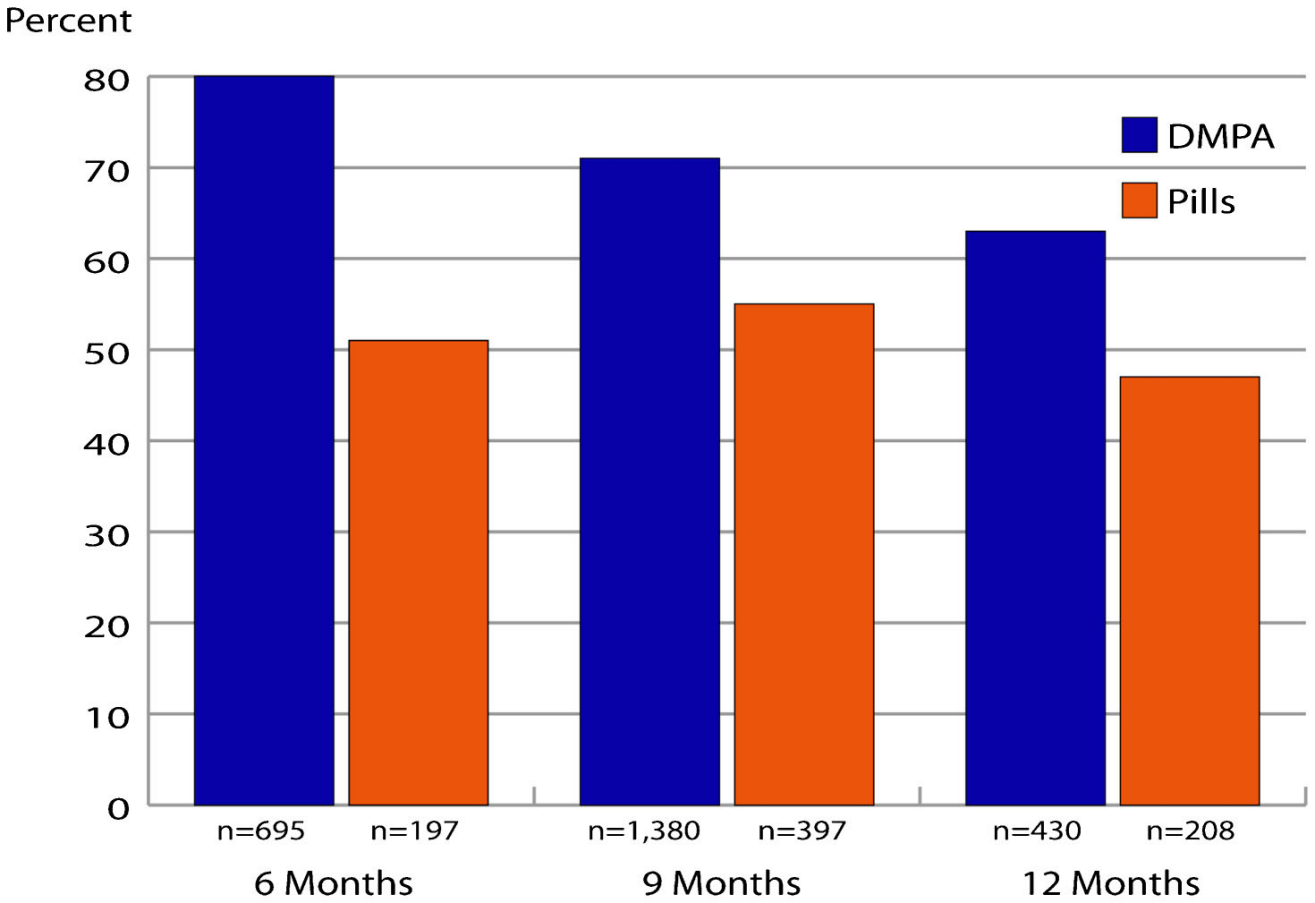
Continued Use of Methods Obtained From CHWs Abbreviations: DMPA, depot medroxyprogesterone acetate. Source: FHI 360 Data Retriever Registers

To determine continuing method use, we examined data from women who obtained DMPA or pills from CHWs during the study who had the opportunity—based on when they started—to use DMPA or pills for 6 months, 9 months, or 12 months (Figure 2). Continuation for DMPA users was always higher than for pill users (using mutually exclusive groups of women with 6, 9, and 12 month follow-up data), with a significant difference noted at the 12-month mark—63% vs. 47% (Chi-square *P*<.001).

### Comparison of District Health Office Statistics with CHW Uptake Data

**Figure 3.**
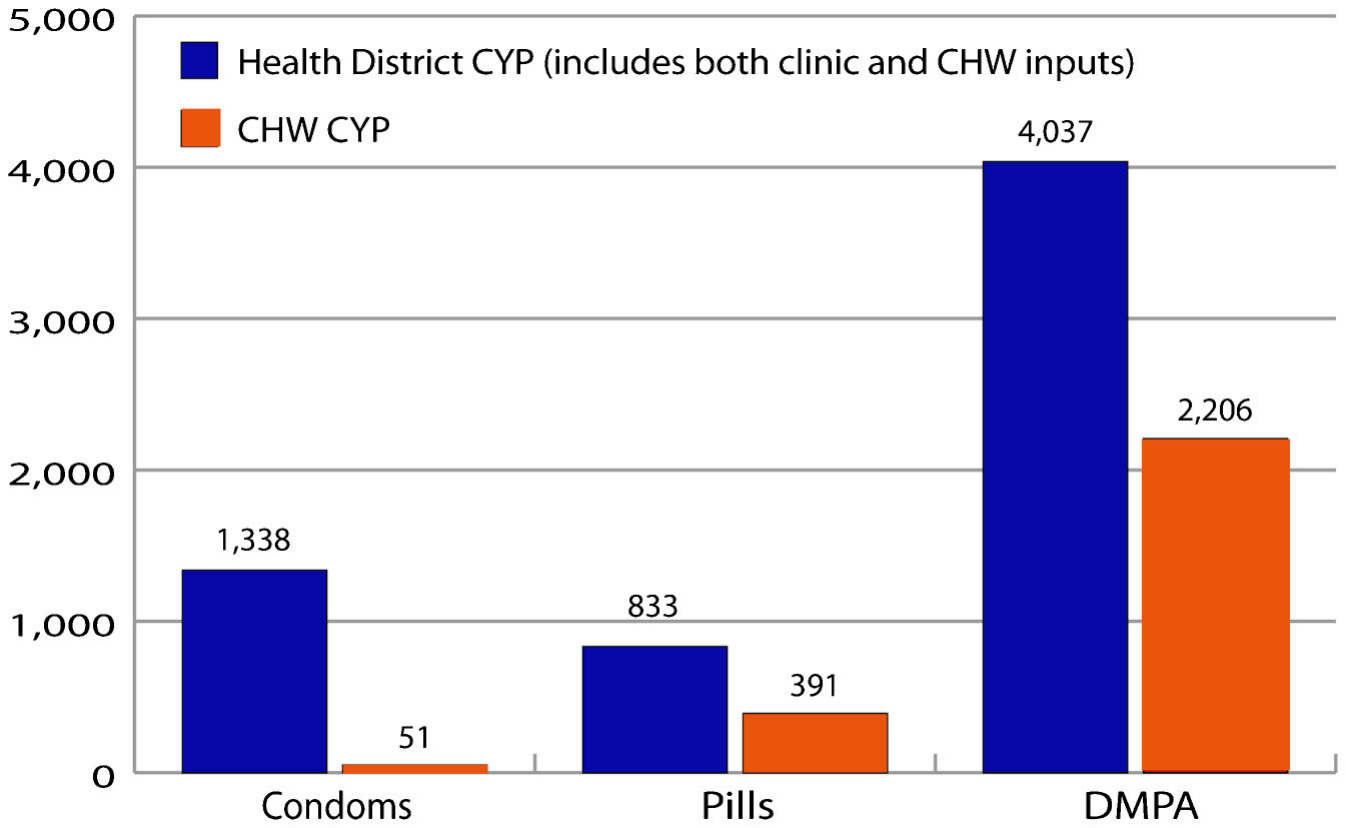
Couple-Years of Protection (CYP), February 2010 to February 2011 Abbreviations: CHW, community health worker; DMPA, depot medroxyprogesterone acetate.

Figure 3 compares CYP data from district-level statistics (which include both clinic and CHW inputs) with records from the ChildFund Zambia CHWs participating in Luangwa and Mumbwa. It is clear from the comparison that many more condoms are provided at the clinic than at the community level, while pill and DMPA provision by the 40 CHWs accounts for about half of the CYPs documented by 34 health centers in Mumbwa and 10 in Luangwa.

### Incremental Cost of Intervention and Per CYP

Table 2 reports incremental costs of intervention activities relevant to future scale up. The total incremental costs were US$37,300 (in 2010 dollars), while the annualized costs adjust some of the cost items to reflect that their effects would extend beyond the initial year of the intervention. For example, the ToT workshop produced 10 persons capable of training CHWs to provide DMPA, but only 4 of them participated in the pilot; therefore, only 40% of the ToT costs are applicable to the pilot. For both training activities, the effects are assumed to last for 2 years (requiring periodic refresher training), and so we applied one-half of the training cost (including practicum expenses) to the 1-year period of service delivery in the pilot program. Other activity costs are unchanged. The estimate of annualized incremental cost (which also serves as the numerator of the incremental cost per CYP ratio) was US$24,322.

**Table 2.** Total and Annualized Incremental Costs of the Intervention (4,600 Zambia Kwacha  =  US$1)

**Intervention Activity**	**Total Cost (US$)**	**Annualized Cost (US$)**
Training of trainers (N = 10)	5,103	1,021
Training of CHWs (N = 40)	12,964	6,482
Practicum expenses	4,827	2,414
Supervision of CHWs	3,334	3,334
Overall intervention management	3,219	3,219
DMPA commodities	7,853	7,853
Total incremental cost^a^	37,300	24,322

^a^As noted, these estimates do not include costs of planning and design, because these are one-time activities that would not be repeated in scale up.

Abbreviations: CHW, community health worker; DMPA, depot medroxyprogesterone acetate.

**Figure 4.**
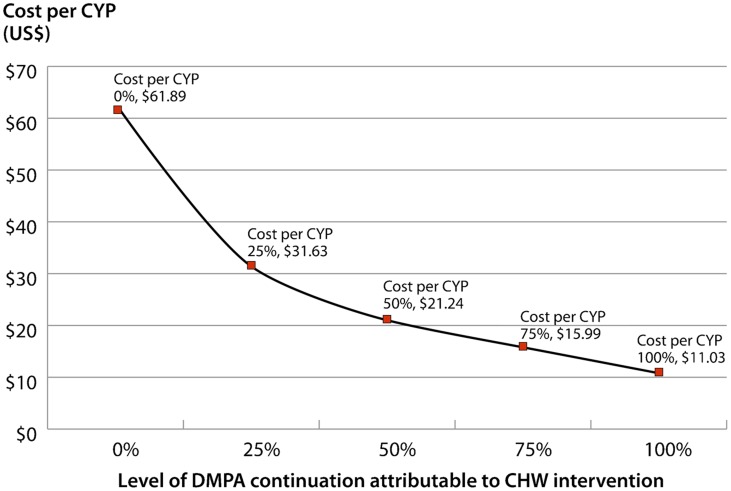
Cost Per Couple-Year of Protection (CYP) at Different Levels of DMPA Continuation Attributable to CHW Intervention Abbreviations: CHW, community health worker; DMPA, depot medroxyprogesterone acetate.

The denominator of the cost per CYP ratio reflects the number of CYPs that can be attributed to the intervention. All CYPs (373) from new DMPA acceptors are included, along with 20 additional CYPs that represent increased contraceptive protection contributed by women who switched to DMPA from the less effective pills and condoms. The remaining CYPs (1,813) can be attributed to the intervention if we assume that these continuing users would not have returned to the clinic for DMPA services. Figure 4 shows the cost per CYP that can definitively be attributed to CHW provision (US$61.89) and the change in cost per CYP at different levels of DMPA continuation. If we assume that 50% of existing DMPA users continued with their method solely because of the improved access afforded by CHW provision, cost per CYP would be lower, at US$21.24. If all users continued because of CHW provision, cost per CYP would be US$11.03.

## DISCUSSION

### Safe, Feasible, and Acceptable

This pilot study contributes to the body of evidence on CHW provision of injectable contraception. As detailed in the Results section of this article, the findings establish the safety, feasibility, and acceptability of CHWs providing DMPA in the Zambian context.

### Impact on Method Use and Choice

The findings also demonstrate the impact of providing DMPA through CHWs on method use and choice, namely that a sizable number of women became new acceptors of all the methods provided by CHWs. Women were also able to switch to a more desirable method and/or service delivery setting.

### Other Factors Affecting Uptake

The project cannot take full credit for the increase in DMPA use in the 2 districts following initiation of the pilot study since the method was also available at 44 health clinics. However, it was a noteworthy increase, made all the more striking by the fact that provision by the 40 CHWs accounted for more than half of the CYPs reported by the District Health Offices from February 2010 to February 2011. This proportion reflects a shortage of health center workers, who were outnumbered by the CHWs, but also points to a pent-up demand for DMPA and women's desire for easier access to this very popular method.

### Additional Benefits of CHW Provision

The benefits of serving clients at the community level go beyond the increase in DMPA use. For example, it can lighten the burden in health centers, leaving nurses more time to provide services that require a higher level of training. For their part, CHWs can learn new skills and build capacity by bringing expanded family planning services to the community. For clients, the reduction in travel and wait times and better access to family planning, including DMPA, can result in fewer women lost to follow-up. Indeed, the use of CHW-provided DMPA at 12 months in this study is significantly higher than that for oral contraceptive pills and is slightly higher than the worldwide norm: typical 1-year continuation rates for DMPA (and pills) are usually between 50% and 60%.^12^

Community-based provision of DMPA allows health center personnel more time to provide services at their higher level of training.

### Cost

Since, as stated above, we do not know the true impact of CHW provision on continuing use of DMPA among family planning users who initiated at clinics, we cannot say with certainty whether the incremental cost per CYP is closer to US$61 or to US$11. Our results showed that cost per CYP declines rapidly with small increases in the proportion of users for whom the convenience of CHW resupply improves DMPA continuation. If we apply the 12-month proportion of continuing DMPA use for CHW-initiated clients (63%), this would suggest an incremental cost per CYP of approximately US$16. Is US$16 per CYP indicative of good value for money? This is difficult to gauge, because comparative data are lacking on cost per CYP for DMPA delivery in other contexts. A recent study in Kenya^13^ estimated facility-based cost per CYP for injectables at US$8.55 to $12.69. Considering that ChildFund's CHW program is reaching women who might not otherwise attend facilities, a US$4 to $8 premium per CYP for bridging the access barrier could be considered good use of scarce resources.

High continuation rates suggest the costs of the intervention can be low—good news for program scale up.

### Indications of Future Demand

Prospects for increasing CHW-initiated and resupplied DMPA in Zambia are very promising. (For a description of the scale-up work already underway in Zambia, see the box.) Once the word spread that certain CHWs were providing DMPA, women flocked to them for the method, including residents of Nangoma, Mumbwa, where the health center is affiliated with the Catholic Church and family planning services are not provided. In Luangwa, some women (not included in our data capture) came to our trained CHWs from neighboring Zimbabwe and Mozambique, since the GoZ provides these services free-of-charge to anyone. Therefore, it was easy for CHWs to find women who wanted to use DMPA.

**BOX. From Research to Program Implementation**

**Strong Stakeholder Leadership**

In May 2011, the Zambian MOH convened a meeting to present the results of the study on the safety, feasibility, and acceptability of DMPA provision by CHWs, to discuss the implications of the results, and to chart the way forward. The FPTWG had helped sustain interest in the pilot study by disseminating progress reports, acting as liaison between the research team and the MOH, and helping to reduce obstacles throughout study implementation. The MOH agreed to continue service delivery in the pilot districts without interruption, to revise the National Health Policy to allow provision of DMPA by CHWs, and to develop a “Road Map for National Scale Up” document. “The Road Map,” drafted by the MOH and partners, was endorsed by a larger group of stakeholders in October 2011.

With support from USAID/Zambia, preparations for the first phase of scale up began in October 2011, with activities beginning in January 2012 that included continued service delivery in the pilot sites, expansion to new sites within the pilot districts, and expansion to new sites in 1 new district (Nyimba). FHI 360 and ChildFund conducted qualitative and quantitative monitoring and evaluation activities, provided technical assistance to the MOH and the Ministry of Community Development, Maternal & Child Health, and facilitated dialogue among stakeholders regarding the policy change to permit CHWs to administer DMPA. In the early scale-up phase, 72 CHWs were newly trained to administer DMPA and now are providing family planning services to their communities.

Ministry-level support for scaling up community-based provision of DMPA was very strong in the immediate post-study phase. This was due in large part to deliberate efforts to engage key stakeholders and influence decision makers from the earliest stages of the research process in 2009 and to maintain that engagement throughout the entire study. Without such concerted efforts to involve stakeholders from the beginning of the study—and the FPTWG's pivotal role in recommending policy changes and scale up in Zambia—it is unlikely that the translation of this study's findings into practice would have occurred so rapidly.

**Study Tour to Sustain Momentum**

However, the general elections immediately following the October 2011 stakeholder meeting led to changes in leadership within the MOH and the creation of a new Ministry of Community Development, Mother & Child Health. These changes decelerated the momentum of the early scale-up process. FHI 360, ChildFund Zambia, and USAID/Zambia worked to orient new leadership to the project and cultivate a renewed sense of ownership. As part of these efforts, a delegation of Zambian stakeholders traveled to Rwanda for a south-to-south tour to observe Rwanda's robust community-based family planning program and engage with stakeholders around important policy-level and operational issues. The study tour effectively improved country-level ownership of the replication process and allowed professional bodies, donors, implementers, and key personnel from both ministries to learn from Rwanda's experience and develop plans for moving ahead with CHW provision of DMPA in Zambia. Many stakeholders are strongly advocating a policy change in 2013.

**Replicability to Other Settings**

It should be noted that this pilot study was implemented within an established CHW program, operated by an NGO that already had a family planning program in place, and with clients who actively sought these services from their CHWs. As such, the intervention was carried out in what could be considered an ideal setting. For that reason, our positive results may not be replicable to the same degree, especially if similar conditions and political will are absent. Nevertheless, this pilot demonstrated: 1) the value of investing in a program where the need for DMPA is ably addressed by a trained cadre of lower-level family planning providers, and 2) the successful expansion of CHW provision of DMPA through effective and continuous collaboration of research, practice, and advocacy.

**Sustaining Commitment**

In Zambia, the potential for this practice to be widely replicated and sustainable is increased in part by a resurgent global interest in family planning. Country-level commitments and support arose out of the highly visible 2012 London Summit on Family Planning. Among them, the GoZ pledged to increase contraceptive prevalence through various strategies, including reducing barriers to task sharing and doubling budget allocations for family planning. Most recently at the 2013 Women Deliver conference, the First Lady of Zambia, Her Excellency Dr. Christine Kaseba-Sata, emphasized her commitment to creating a supportive environment for task sharing and ensuring the scale up of CHW provision of family planning, including DMPA. Scale up has already begun in 3 districts with USAID/Zambia funding and is expected to continue, with appropriate adaptations that will facilitate large-scale expansion, especially as government ownership and funding increase.

## LIMITATIONS OF THE STUDY

As is often the case, family planning statistics obtained at the district level may be incomplete, but to the best of our knowledge all health centers in the districts were included. Nevertheless, it is possible that not all clinics and all CHWs reliably and consistently submitted their monthly forms to the DHO for the time period under study. As such, the data may under-represent the true contributions of clinics and/or CHWs who were not involved in the pilot.

## Footnotes

* The training materials used in 2009 can be accessed at: http://www.k4health.org/toolkits/cba2i/sample-community-health-worker-training-curriculum. However, the content has since been updated to emphasize dual protection to prevent STIs/HIV and a 13-week DMPA re-injection window. Originally the Zambia MOH was following a 12-week policy.

† The FPTWG comprises all local stakeholders interested/involved in family planning, including the Zambia MOH, UNFPA, WHO, USAID, JSI (John Snow, Inc.), ZISSP (Zambia Integrated Systems Strengthening Program), Marie Stopes International, PPAZ (Planned Parenthood Association of Zambia), CHAZ (Churches Health Association of Zambia), World Vision, CDC, SFH (Society for Family Health), and Boston University.

‡ CYP is the estimated protection provided by contraceptive methods during a 1-year period. Our estimates were based on USAID conversion factors for units of condoms (120 per CYP) and pill packs (15 cycles per CYP) distributed, and DMPA injections received (4 doses per CYP). CYPs are not routinely calculated by ChildFund and were computed solely to meet the objectives of the study.

§ During the data collection period, no women stopped and restarted a method after 6 months; we did not determine upon recruitment if continuing users entering the study had restarted a method after 6 months.
